# Extreme βHCG levels in first trimester screening are risk factors for adverse maternal and fetal outcomes

**DOI:** 10.1038/s41598-023-28561-9

**Published:** 2023-01-21

**Authors:** Sarang Younesi, Laleh Eslamian, Nikta Khalafi, Mohammad Mahdi Taheri Amin, Pourandokht Saadati, Soudabeh Jamali, Payam Balvayeh, Mohammad-Hossein Modarressi, Shahram Savad, Saloomeh Amidi, Saeed Delshad, Fariba Navidpour, Bahareh Yazdani, Fatemeh Aasdi, Samira Chagheri, Yalda Mohammadi, Vajiheh Marsoosi, Ashraf Jamal, Soudeh Ghafouri-Fard

**Affiliations:** 1 Nilou Laboratory, Tehran, Iran; 2 Tehran University of Medical Sciences, Tehran, Iran; 3 Department of Medical Genetics, School of Medicine, Tehran University of Medical Sciences, Tehran, Iran; 4 Department of Medical Genetics, Shahid Beheshti University of Medical Sciences, Tehran, Iran

**Keywords:** Biochemistry, Genetics, Biomarkers

## Abstract

Multiples of the normal median (MoM) of free βHCG is a valuable parameter in evaluation of risk of adverse pregnancy outcomes. In the current retrospective study, we assessed the maternal and fetal outcomes in pregnant women having free βHCG MoM levels < 0.2 or > 5 in their first trimester screening (FTS). Relative risk of trisomy 21 was significantly higher in patients having free βHCG MoM > 5. On the other hand, relative risk of trisomies 13 and 18 and Turner syndrome were higher in those having free βHCG MoM < 0.2. Other chromosomal abnormalities were nearly equally detected between those having free βHCG MoM < 0.2 or > 5. Relative risk of hydrocephaly and hydrops fetalis was higher when free βHCG MoM was below 0.2. On the other hand, relative risk of low birth weight was higher when free βHCG MoM was above 5. Moreover, frequency of gestational diabetes mellitus, preeclampsia, preterm delivery and vaginal bleeding increased with levels of free βHCG MoM. However, polyhydramnios had the opposite trend. Frequencies of premature rupture of membranes and pregnancy induced hypertension were highest among pregnant women having levels of free βHCG MoM < 0.2. The current study indicates importance of free βHCG MoM in identification of at-risk pregnancies in terms of both fetal and maternal outcomes. In fact, βHCG MoM < 0.2 or > 5 can be regarded as risk factors for adverse maternal or fetal outcomes irrespective of the presence of other abnormalities in the FTS results.

## Introduction

Maternal serum levels of free beta human chorionic gonadotrophin (βHCG) have been regarded as markers for evaluation of the presence of chromosomal abnormalities in the fetus for a long time^1,2^. Maternal serum levels of free β-hCG has been found to be increased in the first trimester of pregnancy in trisomy 21, while being decreased in trisomies 18 and 13^3,4^. Moreover, this serum marker has been shown to be increased in pregnancies with established preeclampsia and in the third trimester prior to clinical manifestations, yet being decreased or unaltered at 11–13 weeks of gestation^5–8^. Thus, along with assessment of PAPP-A, evaluation of serum levels of free βHCG can enhance the prediction of preterm preeclampsia^9^.

Besides, extreme levels of free βHCG in the first trimester have been correlated with increased frequencies of adverse perinatal outcomes, including intrauterine growth restriction (IUGR) and low birth weight (LBW)^10^. Another large-scale study has approved association between first-trimester free βHCG levels higher than 95th percentile and fetal loss after 24 weeks of gestation^11^. More importantly, low maternal serum βHCG levels have been recently found to be associated with increased risk of having pathogenic findings in chromosomal microarray analysis^12^.

Our recent study in a large cohort of Iranian pregnant women came for amniocentesis has led to identification of the importance of free βHCG multiples of the normal median (MoM) levels < 0.2 or > 5 as indicators of adverse pregnancy outcomes^13^. Thus, we designed the current retrospective study to assess the maternal and fetal outcomes in pregnant women having these free βHCG MoM levels in their first trimester screening (FTS). The current study aimed at investigation of the association between extreme values of βHCG MoM level in FTS and adverse obstetric outcomes. Since fetal sex can affect maternal and obstetric outcomes^14^ as well as βHCG levels^15^, we assessed association between fetal sex and these factors in our study.

The findings of our study can be used for prediction of risk of trisomies as well as pregnancy complications such as preeclampsia, small for gestational age (SGA), gestational diabetes mellitus (GDM) and premature rupture of membranes (PROM).

## Methods

### Patients

In this retrospective study, we assessed data of 327,291 pregnant women referred for FTS to Nilou Laboratory, Tehran, Iran during 2018–2020. Multiple pregnancies were excluded from the study. FTS was based measurement of levels of pregnancy-associated plasma protein-A (PAPP-A) and free βHCG in the maternal blood as well as an ultrasound exam to measure the size of nuchal translucency (NT). The time at which patients referred was between 11 weeks of gestation and 13 weeks and 6 days.

This study included 832 patients who had free βHCG MoM levels < 0.2 and 1447 patients with free βHCG MoM levels > 5 in their FTS assessment. A total of 7494 pregnant women with 0.2 < free βHCG MoM levels < 5 were also included in this study as controls. All cases and controls were Persians. We called all women via phone call in a period of 8–12 months after delivery and collected all relevant data regarding maternal and foetal outcomes. Data was collected in a standardized proforma which was filled by trained staff. Hospital records were also assessed in cases that had abnormal findings. The study protocol was confirmed by the host ethical committee. Maternal demographic features, the results of first trimester sonography and biochemical parameters of all pregnant women at 11 to 13 + 6 weeks of gestation were collected. NT thickness and the presence/absence of nasal bone were also recorded. Serum levels of β-hCG and PAPP-A were assessed by immunofluorescence technique (Delfia Express System; Perkins-Elmer; USA) and used for assessment of risk of trisomies. Levels of serum markers were converted to multiples of the median (MoM) for adjustment of errors associated with race, gestational age, maternal demographic data and mode of conception. The study protocol was approved by Ethical Committee of Shahid Beheshti University of Medical Sciences and all methods were performed in accordance with the relevant guidelines and regulations. Informed written consent forms were signed by all study participants.

Adverse fetal outcomes were defined as follow: stillbirth is a fetal death that occurs at or greater than 24 weeks gestation; intrauterine fetal demise is a fetal death before 23 weeks gestation; fetal morbidity included all problems that have been seen during the newborn period—the first 28 days of life (excluding respiratory problems and jaundice that were reported separately). These problems include seizure, hypoglycemia and infections, any cardiac disorders, grades 3 and 4 intraventricular hemorrhage (associated with cerebral palsy), and vision and hearing impairment (partial or total).

Preterm delivery was defined as spontaneous delivery before 37 weeks of pregnancy.

Respiratory problems included all types of breathing problems that affect newborns, such as transient tachypnea of the newborn, neonatal respiratory distress syndrome (RDS), bronchopulmonary dysplasia (BPD), meconium aspiration syndrome, persistent pulmonary hypertension of the newborn, pneumonia, and apnea.

Death after delivery include death during the newborn period (the first 28 days of life) for any reason. Large and small for gestational age were defined as birth weight above the 90th percentile and below the 10th percentile, respectively.

### Statistical methods

SPSS version 21 (IBM corporation, Armonk, NY, USA) was used for assessment of data. Chi-2 test was used for assessment of association between categorical variables. The relationship between quantitative variables was evaluated using ANOVA test. P values less than 0.05 were considered as significant. Tukey test was used as a nonparametric equivalent for ANOVA for comparison of two or more subgroups within each group. This test was used for comparison of median values of circulatory markers.

## Results

### General information about included cases and controls

The results of comparison of means showed no significant difference between groups in terms of gestational age (P value = 0.238). Yet, there was significant difference in weigh and age of patients within these groups (P values = 0.047 and 0.048, respectively) (Table 1).

**Table 1:** Mean of gestational age, weight and maternal age in different groups of patients based on the levels of free βHCG MoM.

free βHCG MoM		Gestational age (day)	P value	Weight	P value	Age	P value
< 0.2	Mean	75.3	0.238	69.1	0.047	31.2	0.048
	N	832		820		832	
	Std. deviation	3.8		13.8		5.8	
0.2–5	Mean	75.5		75.9		33.8	
	N	7494		2394		7521	
	Std. deviation	3.6		14.9		6.4	
> 5	Mean	75.3		70.5		30.6	
	N	1447		1091		1243	
	Std. deviation	3.6		31.0		5.7	
Total	Mean	75.4		73.2		33.2	
	N	9773		4305		9596	
	Std. deviation	3.6		27.8		6.4	

Totally, 19.6% of pregnant women had a consanguineous marriage. This rate was not different between different groups of women categorized based on the levels of free βHCG MoM (P value > 0.05, Table S1).

In our cohort of patients referred for first trimester screening, 0.25% and 0.44% of all patients had free βHCG MoM < 0.2 and > 5, respectively (Table S2). Most notably, all of patients categorized in the former group were considered as high risk in terms of trisomy 18. However, when categorizing these patients based on risk of trisomy 21, they were nearly equally distributed among high, intermediate and low risk groups. On the other hand, those having βHCG MoM > 5 were considered as high risk for trisomy 21, but this was not true for trisomy 18.

### Frequency of chromosomal abnormalities in different groups of patients

Then, we compared frequencies of chromosomal abnormalities between different groups of patients based on the levels of free βHCG MoM (Table 2). Based on the results of Fisher exact test, levels of free βHCG MoM were associated with frequencies of chromosomal abnormalities (P < 0.001). Relative risk of trisomy 21 was significantly higher in patients having free βHCG MoM > 5. On the other hand, relative risk of trisomies 13 and 18 and Turner syndrome were higher in those having free βHCG MoM < 0.2. Other chromosomal abnormalities were nearly equally detected between those having free βHCG MoM < 0.2 or > 5.

**Table 2:** Frequency of chromosomal abnormalities in different groups of patients based on the levels of free βHCG MoM (RR, relative risk).

		free βHCG MoM			P value
		< 0.2	0.2–5	> 5	
Trisomy 21	Count	7	222	24	P < 0.001
	% within free βHCG MoM categories	6.8%	71.6%	82.8%	
	RR	0.09	1	1.16	
Trisomy 18	Count	58	35	0	P < 0.001
	% within free βHCG MoM categories	56.4%	11.3%	0.0%	
	RR	4.99	1	0	
Trisomy 13	Count	26	19	0	P < 0.001
	% within free βHCG MoM categories	25.2%	6.1%	0.0%	
	RR	4.13	1	0	
Turner Syndrome	Count	5	17	0	P > 0.05
	% within free βHCG MoM categories	4.9%	5.5%	0.0%	
	RR	0.89	1	0	
Klinefelter Syndrome	Count	1	4	0	P > 0.05
	% within free βHCG MoM categories	0.9%	1.3%	0.0%	
	RR	0.69	1	0	
Other chromosomal abnormalities	Count	6	13	5	P > 0.05
	% within free βHCG MoM categories	5.8%	4.2%	17.2%	
	RR	1.38	1	4.09	
Total	Count	103	310	29	–
	% within free βHCG MoM categories	100.0%	100.0%	100.0%	

### Adverse fetal/maternal outcomes

We also compared the frequencies of other neonatal complications between different groups of pregnant women based on the levels of free βHCG MoM (Table 3). Fisher exact test showed significant associations between these complications and levels of free βHCG MoM (P < 0.001). Relative risk of hydrocephaly and hydrops fetalis was higher when free βHCG MoM was below 0.2. On the other hand, relative risk of low birth weight was higher when free βHCG MoM was above 5.

**Table 3:** Frequencies of other neonatal complications between different groups of pregnant women based on the levels of free βHCG MoM (RR, relative risk).

		free βHCG MoM			Total
		< 0.2	0.2–5	> 5	
Fetal morbidity	Count	3	117	18	138
	% within free βHCG MoM categories	4.7%	24.9%	18.4%	
	RR	0.19	1	0.74	
	P-value	< 0.001		0.164	
Stillbirth	Count	1	10	3	14
	% within free βHCG MoM categories	1.6%	2.1%	3.1%	
	RR	0.76	1	1.48	
	P-value	0.764		0.576	
Respiratory problems	Count	3	42	10	55
	% within free βHCG MoM categories	4.7%	9.0%	10.2%	
	RR	0.52	1	1.13	
	P-value	0.249		0.697	
Death after delivery	Count	0	8	4	12
	% within free βHCG MoM categories	0.0%	1.7%	4.1%	
	RR	0	1	2.41	
	P-value			0.137	
Hydrocephaly	Count	3	4	0	7
	% within free βHCG MoM categories	4.7%	0.8%	0.0%	0.5%
	RR	5.87	1	0	
	P-value	0.011			
Hospitalization	Count	5	17	8	30
	% within free βHCG MoM categories	7.8%	3.6%	8.2%	2.3%
	RR	2.17	1	2.28	
	P-value	0.114		0.047	
Hydronephrosis	Count	0	5	0	5
	% within free βHCG MoM categories	0.0%	1.0%	0.0%	0.4%
	RR	0	1	0	
	P-value	-	-	-	
Intrauterine fetal demise	Count	10	87	16	113
	% within free βHCG MoM categories	15.6%	19.0%	16.3%	8.8%
	RR	0.82	1	0.86	
	P-value	0.569		0.604	
Structural Anomaly	Count	30	136	20	186
	% within free βHCG MoM categories	23.4%	29.0%	20.4%	14.4%
	RR	0.81	1	0.70	
	P-value	0.004		0.083	
Cystic Hygroma	Count	1	1	0	2
	% within free βHCG MoM categories	1.6%	0.2%	0.0%	0.2%
	RR	8.0	1	0	
	P-value	0.226			
Skeletal Dysplasia	Count	1	4	0	5
	% within free βHCG MoM categories	1.6%	0.8%	0.0%	0.4%
	RR	2.0	1	0	
	P-value	0.581			
Low birth weight	Count	2	19	15	32
	% within free βHCG MoM categories	3.2%	4.0%	15.3%	2.5%
	RR	0.8	1	3.82	
	P-value	0.721		< 0.001	
Hydrops fetalis	Count	5	4	0	9
	% within free βHCG MoM categories	7.8%	0.8%	0.0%	0.7%
	RR	9.75	1	0	
	P-value	< 0.001			
Nuchal Cord	Count	0	8	0	1
	% within free βHCG MoM categories	0.0%	1.7%	0.0%	0.1%
	RR	0	1	0	
	P-value	–	–	–	
Omphalocele	Count	0	0	4	4
	% within free βHCG MoM categories	0.0%	0.0%	4.0%	0.3%
	RR	0	0	–	
	P-value	–	–		
Intrauterine growth restriction	Count	0	7	0	3
	% within free βHCG MoM categories	0.0%	1.4%	0.0%	0.2%
	RR	0	1	0	
	P-value	–	–	–	
Total	Count	64	469	98	616
	% within free βHCG MoM categories	100.0%	100.0%	100.0%	100.0%

Similarly, Fisher exact test showed significant association between adverse maternal outcomes and levels of free βHCG MoM (P < 0.001). Frequency of GDM, preeclampsia, preterm delivery and vaginal bleeding increased with levels of free βHCG MoM. However, polyhydramnios had the opposite trend. Frequencies of PROM and pregnancy induced hypertension (PIH) were highest among pregnant women having levels of free βHCG MoM < 0.2 (Table S3).

High risk for each trisomy was considered as having risk > 1/250 in FTS. Intermediate risk was defined as having 1/1500 < risk < 1/251 for trisomy 21 and 1/1000 < risk < 1/251 for trisomies 13 and 18. Finally, low risk was defined as risk < 1/1500 for trisomy 21 and risk < 1/1000 for trisomies 13 and 18.

Kruskal–Wallis test and Tukey test for intra-group comparisons showed significant differences in odds of being affected given a positive result (OAPR) in high risk groups (P < 0.001) (Table 4). In fact, in high risk group when free βHCG levels are below 0.2, one out of 3.7 pregnant women referred for diagnostic procedures has a pathologic chromosomal finding. These ratios are 1/16.7 and 1/25.4 in high risk groups with free βHCG levels of 0.2–5 and > 5, respectively.

**Table 4:** Distribution of chromosomal abnormalities, structural anomalies, adverse fetal/maternal outcomes in different groups of pregnant women based on the levels of free βHCG MoM and calculated risk in first trimester screening (HR, high risk; IR, intermediate risk; LR, low risk).

FBhCG MoM	Risk group	Number	T21 number (%)	T18 or T13 number (%)	Other chromosomal abnormalities	Structural anomaly	Fetal adverse outcomes	Maternal adverse outcomes	Total adverse outcomes*	OAPR (total)**
< 0.2	HR	315	4 (1.27%)	72 (22.9%)	10 (3.17%)	22 (6.98%)	30 (9.52%)	51 (16.2%)	77 (24.4%)	1:3.7
	IR	229	2 (0.87%)	8 (3.5%)	5 (2.2%)	6 (2.6%)	14 (6.1%)	26 (11.4%)	27 (11.8%)	1:16.3
	LR	288	1 (0.35%)	4 (1.4%)	2 (0.69%)	3 (1.04%)	14 (4.86%)	19 (6.6%)	26 (9.02%)	1:41.3
Total		832	6 (0.72%)	84 (10.1%)	17 (2.04%)	31 (3.73%)	58 (6.97%)	95 (11.4%)	130 (15.6%)	1:7.7
0.2–5.0	HR	4731	208 (4.4%)	49 (1.04%)	27 (0.57%)	122 (2.58%)	347 (7.33%)	409 (8.65%)	606 (12.81%)	1:16.7
	IR	829	14 (1.69%)	4 (0.48%)	3 (0.36%)	10 (1.21%)	52 (6.27%)	61 (7.36%)	89 (10.7%)	1:39.5
	LR	1934	1 (0.05%)	1 (0.05%)	1 (0.05%)	6 (0.31%)	80 (4.14%)	103 (5.33%)	164 (8.48%)	1:644.7
Total		7494	223 (2.98%)	54 (0.72%)	31 (0.41%)	138 (1.84%)	479 (6.39%)	573 (7.65%)	859 (11.46%)	1:24.3
> 5.0	HR	507	18 (3.55%)	0	2 (0.4%)	18 (3.55%)	41 (8.11%)	56 (11.1%)	75 (14.8%)	1:25.4
	IR	524	6 (0.95%)	0	2 (0.19%)	2 (0.19%)	40 (7.63%)	50 (9.54%)	67 (12.7%)	1:65.5
	LR	416	0	0	1 (0.67%)	1 (0.67%)	27 (6.5%)	33 (7.93%)	45 (10.8%)	1:416
Total		1447	24 (1.66%)	0	5 (0.35%)	21 (1.45%)	108 (7.46%)	139 (9.6%)	187 (12.9%)	1:49.9

*Indicates the presence of at least one fetal/maternal adverse outcome.

**Odds of being affected given a positive result.

When free βHCG levels are below 0.2, risk of trisomies 13 and 18 and other chromosomal abnormalities increase in all three categories of high, intermediate and low risks based on the results of FTS (P < 0.001). If we include all chromosomal anomalies in the analyses in this group, an OAPR value of 1/7.7 is calculated (107 chromosomal abnormalities in a total of 832 cases).

Besides, risk of structural abnormalities is higher in patients having free βHCG levels are below 0.2 (P < 0.001). Moreover, both adverse fetal outcomes and adverse maternal outcomes (including preterm delivery, PROM, preeclampsia, vaginal bleeding, amniotic leakage and PIH) are higher in patients having free βHCG levels are below 0.2.

In pregnancies with free βHCG levels between 0.2 and 5, OAPR was different between intermediate and low risk groups (P < 0.001). OAPR was calculated to be 1/39.5 in this group.

Finally, free βHCG MoM > 5 has importance only in high risk group. In the intermediate risk group, an OAPR value of 1/65.5 was obtained which is not appropriate.

We also assessed association between adverse fetal outcomes and calculated risk of trisomy 18 (Table S4). Fisher exact test showed association between averse fetal outcomes and calculated risk of trisomy 18 (P < 0.001). Frequencies of IUFD, structural anomalies and skeletal dysplasia increased with increasing risk of trisomy 18. Similar results were obtained for trisomy 21 (data not shown).

Moreover, risks of PROM, abortion, preeclampsia and HELLP syndromes were increased with increased risks of trisomies 18 and 21.

### Association between fetal sex and chromosomal abnormalities or adverse pregnancy outcomes

Frequencies of trisomies 21 and 18 were significantly higher in male fetuses (Table S5).

PROM, fetal morbidity, stillbirth, hospitalization and LBW were higher in female fetuses. On the other hand, gestational diabetes mellitus, PIH, spontaneous abortion, IUFD and structural had higher frequencies in male fetuses (Table 5).

**Table 5:** Association between fetal sex and adverse fetal/maternal outcomes.

		Sex		Total
		Female	Male	
Fetal morbidity	Count	52	33	85
	% within sex	12.7%	8.8%	10.8%
Still birth	Count	9	1	10
	% within sex	2.2%	0.3%	1.3%
Hospitalization	Count	14	5	19
	% within sex	3.4%	1.3%	2.4%
IUFD	Count	8	23	31
	% within sex	2.0%	6.1%	4.0%
Structural anomalies	Count	56	68	124
	% within sex	13.7%	18.1%	15.8%
Low birth weight	Count	16	7	23
	% within sex	3.9%	1.9%	2.9%
Total	Count	409	375	784
	% within sex	100.0%	100.0%	100.0%

## Discussion

In our recent study, we have suggested high risk non-invasive prenatal testing (NIPT), PAPP-A MoM < 0.2 and free βHCG MoM > 5 or < 0.2 as important indications for necessity of invasive testing in pregnancy with appropriate OAPR values^13^. In the current study, we have collected data about perinatal complications and adverse fetal/maternal outcomes from patients with free βHCG MoM > 5 or < 0.2 in a period of 8–12 month after delivery.

It is worth mentioning that evaluation of serum levels of βHCG and other biochemical factors in each ethnic group has practical significance. This supposition is supported by previous studies. For instance, Chinese women have been shown to have higher maternal serum levels of free βHCG and PAPP-A in the first trimester, after correction for the maternal weight^16^. Similarly, significant differences have been observed in European and Asian MoM values^17^. Thus, adjustment for ethnicity is important in FTS programs.

We found that relative risk of trisomies 13 and 18 and Turner syndrome were higher in those having free βHCG MoM < 0.2. Relative risk of hydrocephaly and hydrops fetalis was higher when free βHCG MoM was below 0.2. On the other hand, relative risk of low birth weight was higher when free βHCG MoM was above 5. Moreover, frequency of GDM, preeclampsia, preterm delivery and vaginal bleeding increased with levels of free βHCG MoM. However, polyhydramnios had the opposite trend. Frequencies of premature rupture of membranes and pregnancy induced hypertension were highest among pregnant women having levels of free βHCG MoM < 0.2. Thus, levels of this serum marker can predict occurrence a wide array of maternal/fetal adverse outcomes.

From a mechanistic point of view, numerous functions of hCG can be related with these adverse outcomes. For instance, hCG has a role in enhancement of production of progesterone by corpus luteal cells and induction of angiogenesis in uterine vasculature. Moreover, it promotes the fusion of cytotrophoblast cell and differentiation to make syncytiotrophoblast cells. Most importantly, hCG contributes to the blockage of maternal immune or macrophage actions on foreign invading placental cells and causes uterine growth parallel to fetal growth^18^. Thus, it is not surprising that abnormal levels of free βHCG is associated with a wide range of maternal and fetal adverse outcomes. In fact, hCG contributes to the several functional and structural aspects of healthy pregnancy.

In our cohort of patients, the rate of consanguinity was 19.6. Although there is evidence to suggest association between consanguinity and genetic and congenital abnormality as well as adverse pregnancy outcomes^19,20^, this rate was not different between different groups of women categorized based on the levels of free βHCG MoM.

Sharony et al., have detected low maternal serum concentrations of hCG in 0.57% of the assessed women. Approximately, 16% of these women have been found to have missed abortions. They have not detected any case of trisomy 18 or other chromosomal abnormalities in their patient population. Yet, they have reported perinatal complications in these pregnancies^21^.

In the study conducted by Ong et al.^22^, maternal serum free βhCG below 10th centile of the reference range was detected in about 15% of the pregnancies that afterwards led to miscarriage or resulted in pregnancy induced hypertension or growth restriction, and in 20% of pregnancies that developed GDM, indicating the importance of this marker in the prediction of pregnancy complications. In fact, low maternal serum free βhCG below might indicate insufficiency of placenta.

In patients having βHCG levels below 0.2, if we include all chromosomal anomalies in the analyses, an OAPR value of 1/7.7 is calculated. Considering the acceptable value of OAPR for FTS (1/22–1/25, i.e. positive screening results of 4–5%), this OAPR indicates that free βHCG levels < 0.2 can be used as a novel indication for amniocentesis. Since this result is found in 0.25% of total patients, this indication can result in improvement of final OAPR in screening of chromosomal abnormalities. Besides, the observed higher risk of structural abnormalities in patients having free βHCG levels below 0.2 indicates necessity for precise follow-up of these patients using higher standards anomaly scans. Moreover, both fetal and maternal adverse outcomes were higher in patients having free βHCG levels are below 0.2. Thus, these pregnancies should be considered as high risk pregnancies receiving higher levels of care, including follow-up of pregnant women for monitoring blood glucose and blood pressure, exact monitoring of fetal weight and delivery at more specialized centers.

Recently, a large-scale study in Turkish population has reported association between serum levels of PAPP-A and SGA, while GDM, PROM and preterm PPROM have been more common in pregnant women with low serum free β-hCG^23^. The latter finding is in accordance with our finding regarding the higher frequencies of PROM among pregnant women having levels of free βHCG MoM < 0.2. However, we found association between GDM and high levels of free βHCG MoM. There are studies showing lower risk of GDM in pregnancies with high level of βhCG (≥ 2.0 MoM)^24,25^. Two other studies have reported no association between serum free βhCG levels and GDM^26,27^.

Mechanistic studies have indicated that a number of signaling pathways and biomolecules including NF-κB, PPARs, SIRTs, AMPK, GSK3, PI3K/mTOR, inflammasome and endoplasmic reticulum stress are implicated in the pathogenesis of GDM^28^. βhCG has functional interactions with a number of these pathways. For instance, it attenuates NF-κB activation and cytokine expression^29^. Moreover, PI3K/mTOR signaling pathway is involved in the hCG-mediated induction of vascular endothelial growth factor^30^. Although the exact mechanism of contribution of abnormal levels of βhCG in the pathogenesis of GDM is not clear, abnormalities in the regulation of these pathways might contribute to this pathology.

High level of βhCG has been shown to be a predictor of preeclampsia in another study in Iranian pregnant women. In fact, βhCG > 3 MOM has been associated with more than five-fold increase in the risk of developing preeclampsia in the mentioned study^31^. This finding is in accordance with our results.

In pregnancies with free βHCG levels between 0.2 and 5, OAPR was different between intermediate and low risk groups. Since OAPR was calculated to be 1/39.5 in this group, we recommend inclusion of other parameters such as maternal age > 35, fetal NT ≥ 99^th^ or 95^th^ percentile and free βHCG MoM/PAPP-A MoM > 3 in assessment of patients. Based on these parameters, second trimester screening, sequential combined tests or NIPT can be recommended for pregnant women.

Finally, since free βHCG MoM > 5 has importance only in high risk group based on the FTS results, we recommend using the calculated risk of FTS for further assessments. Risk of adverse maternal/fetal outcomes in this category was significantly different from control group in all three high, intermediate and low risk groups indicating the importance of higher care systems for this group of pregnant women.

We also found associations between some parameters of adverse maternal/fetal outcomes and fetal sex as well as calculated risk of trisomies 18 and 21 in FTS which is in line with our previous study^13^.

Future studies are needed to assess the role of other factors such as paternal age, residential area, nutritional deficiency and chemical or biological contamination on the results of FTS and interpretation of βHCG MoM levels. In the current study, we did not assess the possible impact of these factors on the obtained results of FTS. The role of these factors in risk of trisomies is controversial. For instance, while Quattrocchi et al.^32^ have reported no correlation between paternal age and risk of trisomy 21, other studies have reported positive and inverse associations between this factor and risk of trisomy 21, respectively^16,17^.

Taken together, the current study indicates importance of free βHCG MoM in identification of at-risk pregnancies in terms of both fetal and maternal outcomes and proposes extreme βHCG MoM values as indicators of necessity of invasive testing during pregnancy. In fact, βHCG MoM < 0.2 or > 5 can be regarded as risk factors for adverse maternal or fetal outcomes irrespective of the presence of other abnormalities in the FTS results. A possible explanation for association between adverse outcomes and both low and high levels of βHCG is that these extreme values reflect inappropriate function of placenta.

Our study has some limitations. We did not analyze the effects of socioeconomic status in this study. Although our study was conducted in a large group of pregnant women, the results should be confirmed in multicenter prospective studies. Finally, in our study, patients reported outcomes following a telephone call at 8 to 12 months post-delivery. This may not be reliable and potentially subject to recall bias.

## Supplementary Information

Supplementary Tables.

## Data Availability

All data generated or analysed during this study are included in this published article [and its supplementary information files].
