# Supplementary material for: Extreme βHCG levels in first trimester screening are risk factors for adverse maternal and fetal outcomes
Source: Sci Rep. 2023 Jan 21;13:1228. doi: 10.1038/s41598-023-28561-9 (PMC9867730; doi:10.1038/s41598-023-28561-9)
Supplement: Supplementary file 1 — Supplementary Tables. [file 41598_2023_28561_MOESM1_ESM.docx]

Supplementary material

Table S1. Frequency of consanguineous marriage among different groups of patients based on the levels of free βHCG MoM.

|  | | | Marriage | | Total |
| --- | --- | --- | --- | --- | --- |
|  |  |  | Consanguineous | Non-consanguineous |  |
| free βHCG MoM | <0.2 | Count | 196 | 624 | 820 |
|  |  | % within free βHCG MoM categories | 23.9% | 76.1% | 100.0% |
|  | 0.2-5 | Count | 413 | 1953 | 2366 |
|  |  | % within free βHCG MoM categories | 17.5% | 82.5% | 100.0% |
|  | >5 | Count | 229 | 856 | 1085 |
|  |  | % within free βHCG MoM categories | 21.1% | 78.9% | 100.0% |
| Total | | Count | 838 | 3433 | 4271 |
|  |  | % within free βHCG MoM categories | 19.6% | 80.4% | 100.0% |

Table S2. Distribution of patients based on the calculated risks for trisomies 21 and 18 and different levels of free βHCG MoM.

| Risk of T18 (%) | Risk of T21 (%) | Patients |
| --- | --- | --- |
| 1,342 (0.41) | 14,138 (4.3) | High Risk |
| 1,734 (0.53) | 49,584 (15.2) | Intermediate Risk |
| 324,215 (99.06) | 263,569 (80.5) | Low risk |
| 327,291 | 327,291 | Total |

Table S3. Frequency of maternal adverse outcomes in different groups of pregnant women based on the levels of free βHCG MoM.

|  | | free βHCG MoM | | | Total |
| --- | --- | --- | --- | --- | --- |
|  |  | <0.2 | 0.2-5 | >5 |  |
| Gestational diabetes mellitus | Count | 5 | 29 | 10 | 44 |
|  | % within free βHCG MoM categories | 5.1% | 5.1% | 7.0% | 5.4% |
| Premature rupture of membranes | Count | 43 | 127 | 39 | 209 |
|  | % within free βHCG MoM categories | 43.4% | 22.4% | 27.3% | 25.9% |
| Spontaneous abortion | Count | 15 | 111 | 21 | 147 |
|  | % within free βHCG MoM categories | 15.2% | 19.6% | 14.7% | 18.2% |
| Polyhydramnios | Count | 2 | 1 | 0 | 3 |
|  | % within free βHCG MoM categories | 2.0% | 0.2% | 0.0% | 0.4% |
| Preeclampsia | Count | 2 | 69 | 18 | 89 |
|  | % within free βHCG MoM categories | 2.0% | 12.2% | 12.6% | 11.0% |
| Preterm delivery | Count | 30 | 160 | 34 | 224 |
|  | % within free βHCG MoM categories | 15.2% | 17.2% | 21.5% | 2.2% |
| Maternal infection | Count | 0 | 28 | 0 | 28 |
|  | % within free βHCG MoM categories | 0.0% | 4.9% | 0.0% | 3.5% |
| HELLP Syndrome | Count | 0 | 7 | 0 | 7 |
|  | % within free βHCG MoM categories | 0.0% | 1.2% | 0.0% | 0.9% |
| Pregnancy-induced hypertension (PIH) | Count | 8 | 16 | 5 | 29 |
|  | % within free βHCG MoM categories | 8.1% | 2.8% | 3.5% | 3.6% |
| Amniotic leakage | Count | 8 | 37 | 10 | 55 |
|  | % within free βHCG MoM categories | 8.1% | 6.5% | 7.0% | 6.8% |
| Spotting | Count | 15 | 112 | 36 | 163 |
|  | % within free βHCG MoM categories | 15.2% | 19.8% | 25.2% | 20.2% |
| Total | Count | 128 | 697 | 173 | 998 |
|  | % within free βHCG MoM categories | 100.0% | 100.0% | 100.0% | 100.0% |

Table S4. Association between adverse fetal outcomes and calculated risk of trisomy 18.

|  | | Trisomy 18 risk | | | Total |
| --- | --- | --- | --- | --- | --- |
|  |  | <250 | 250-1000 | >1000 |  |
| Fetal morbidity | Count | 1 | 3 | 138 | 142 |
|  | % within trisomy 18 risk categories | 0.3% | 3.6% | 15.7% | 17.3% |
| Stillbirth | Count | 1 | 0 | 13 | 14 |
|  | % within trisomy 18 risk categories | 0.3% | 0.0% | 1.5% | 1.1% |
| Respiratory problems | Count | 2 | 0 | 53 | 55 |
|  | % within trisomy 18 risk categories | 0.6% | 0.0% | 6.0% | 4.3% |
| Death after delivery | Count | 0 | 0 | 12 | 12 |
|  | % within trisomy 18 risk categories | 0.0% | 0.0% | 1.4% | 0.9% |
| Hydrocephaly | Count | 2 | 2 | 3 | 7 |
|  | % within trisomy 18 risk categories | 0.6% | 2.4% | 0.3% | 0.5% |
| Polyhydramnios | Count | 0 | 0 | 1 | 1 |
|  | % within trisomy 18 risk categories | 0.0% | 0.0% | 0.1% | 0.1% |
| Hospitalization | Count | 0 | 2 | 28 | 30 |
|  | % within trisomy 18 risk categories | 0.0% | 2.4% | 3.2% | 2.3% |
| Hydronephrosis | Count | 0 | 0 | 5 | 5 |
|  | % within trisomy 18 risk categories | 0.0% | 0.0% | 0.6% | 0.4% |
| Intrauterine fetal demise | Count | 29 | 4 | 80 | 113 |
|  | % within trisomy 18 risk categories | 8.8% | 4.8% | 9.1% | 8.7% |
| Structural Anomaly | Count | 49 | 20 | 117 | 186 |
|  | % within trisomy 18 risk categories | 14.9% | 23.8% | 13.3% | 14.4% |
| Cystic Hygroma | Count | 2 | 0 | 0 | 2 |
|  | % within t18.cat | 0.6% | 0.0% | 0.0% | 0.2% |
| Skeletal Dysplasia | Count | 5 | 0 | 0 | 5 |
|  | % within trisomy 18 risk categories | 1.5% | 0.0% | 0.0% | 0.4% |
| Low birth weight | Count | 1 | 0 | 31 | 32 |
|  | % within trisomy 18 risk categories | 0.3% | 0.0% | 3.5% | 2.5% |
| Hydrops fetalis | Count | 6 | 0 | 3 | 9 |
|  | % within trisomy 18 risk categories | 1.8% | 0.0% | 0.3% | 0.7% |
| Nuchal Cord | Count | 0 | 0 | 1 | 1 |
|  | % within trisomy 18 risk categories | 0.0% | 0.0% | 0.1% | 0.1% |
| Omphalocele | Count | 0 | 0 | 4 | 4 |
|  | % within trisomy 18 risk categories | 0.0% | 0.0% | 0.5% | 0.3% |
| Intrauterine growth restriction | Count | 0 | 0 | 3 | 3 |
|  | % within trisomy 18 risk categories | 0.0% | 0.0% | 0.3% | 0.2% |
| Total | Count | 98 | 31 | 492 | 621 |
|  | % within trisomy 18 risk categories | 100.0% | 100.0% | 100.0% | 100.0% |

Table S5. Association between fetal sex and chromosomal abnormalities.

|  | | Sex | | Total |
| --- | --- | --- | --- | --- |
|  |  | female | male |  |
| Down syndrome | Count | 75 | 92 | 167 |
|  | % within sex | 18.3% | 24.5% | 21.3% |
| Trisomy 18 | Count | 19 | 28 | 47 |
|  | % within sex | 4.6% | 7.5% | 6.0% |
| Trisomy 13 | Count | 15 | 8 | 23 |
|  | % within sex | 3.7% | 2.1% | 2.9% |
|  | % within sex | 4.9% | 6.4% | 5.6% |
| Other chromosomal abnormalities | Count | 10 | 6 | 16 |
|  | % within sex | 2.4% | 1.6% | 2.0% |
| Total | Count | 409 | 375 | 784 |
|  | % within sex | 100.0% | 100.0% | 100.0% |
